# Phylogenetic Diversity and Geographic Distribution of Atlantic Salmon Calicivirus in Major Salmon Farming Regions

**DOI:** 10.1111/jfd.14107

**Published:** 2025-02-19

**Authors:** Vincenzo A. Costa, Aase B. Mikalsen, Francisca Samsing

**Affiliations:** ^1^ Sydney School of Veterinary Science The University of Sydney Camden New South Wales Australia; ^2^ Faculty of Veterinary Medicine Norwegian University of Life Sciences Ås Norway

**Keywords:** Atlantic salmon, calicivirus, metatranscriptomics

## Abstract

*Salovirus* is a genus within the family *Caliciviridae*, which contains a single member species, *Salovirus nordlandense*, also known as Atlantic salmon calicivirus (ASCV). While previous work has shown that ASCV can replicate in fish cell lines and establish systemic infection in vivo, its exact role in disease remains unclear and very little is known about its geographic distribution and evolution among Atlantic salmon. To expand the phylogenetic range of ASCV and better understand its potential role in disease, we screened publicly available transcriptomes for ASCV‐like sequences. Notably, we detected ASCV in sequencing projects of Atlantic salmon ( *Salmo salar* ) (*n* = 40) and wild common whitefish ( *Coregonus lavaretus* ) (*n* = 1), across Chile, Scotland and Norway. Our phylogenetic analysis identified two viral species, which we provisionally name *Salovirus nordlandense 1* and *2*, each containing distinct genotypes. Both viral species were found in all three countries, with no clear geographic pattern, indicating that saloviruses have spread through the Atlantic salmon trade. It was notable that 88% of these transcriptomes were generated for the study of other pathogens, including infectious salmon anaemia virus, piscine myocarditis virus and *Piscirickettsia salmonis* , suggesting that saloviruses might be frequently associated with co‐infections. Overall, this study indicates that viruses, like ASCV, can silently spread through aquacultural practices, potentially contributing to a variety of fish diseases.

## 1 Introduction

The *Caliciviridae* is a large family of positive‐sense single‐stranded RNA viruses that infect all classes of vertebrates and are classified into 11 genera (Vinje et al. 2019). Despite their characterisation in lower vertebrates (Shi et al. 2018), they have predominantly been studied in mammals, and relatively little is known about their impact on finfish. At present, there are two classified genera that infect finfish, *Salovirus* and *Minovirus* (Mikalsen et al. 2014; Mor et al. 2017), as well as a variety of divergent caliciviruses awaiting classification (Shi et al. 2018). Atlantic salmon calicivirus (ASCV; also known as *Salovirus nordlandense*) represents the first calicivirus to be isolated from diseased finfish and was demonstrated to cause experimental systemic infection in Atlantic salmon ( *Salmo salar* ) in Norway (Mikalsen et al. 2014). In addition, ASCV was also identified in farmed Atlantic salmon and wild chinook salmon ( *Oncorhynchus tshawytscha* ) in Canada (Mordecai et al. 2021; Bateman et al. 2021).

Caliciviruses possess 6.4–8.5 kb genomes encoding a polyprotein, ORF1, and two structural proteins, VP1 and VP2, with the former contiguous with ORF1 at the 3′ end in all genera except for *Norovirus*, *Recovirus* and *Vesivirus* (Vinje et al. 2019). The calicivirus protease protein (NS6) is responsible for the co‐ and post‐translational cleavage of ORF1 into six non‐structural proteins: NS1/2 (p26), NS3 (ATPase), NS4 (p16), NS5 (VPg), NS6, and NS7 (RNA‐dependent RNA polymerase [RdRp]) (Vinje et al. 2019).

Phylogenetic comparisons of the *Caliciviridae* have placed the genus *Salovirus* among other fish caliciviruses, with phylogenetic divergence between fish, mammalian and avian infecting genera (Mordecai et al. 2021; Shi et al. 2018). Based on the limited genomic information available, ASCV is composed of three distinct viruses that may represent different viral species or genotypes (Mordecai et al. 2021). For example, Mikalsen et al. (2014) identified two isolates of ASCV in Atlantic salmon with heart pathology in Norway—one derived from cell culture (AL V901) and another from the field (Nordland/2011). Similarly, in Canada, ASCV was identified in more than 50% of farmed Atlantic salmon, the vast majority of which were moribund (Mordecai et al. 2021).

To better understand the diversity and evolution of ASCV among Atlantic salmon, and the risk of disease to the global salmon industry, we screened for ASCV using published RNA sequencing data from NCBI databases. Using these data, we aimed to expand the phylogenetic and geographic diversity of ASCV and reveal its distribution among farmed Atlantic salmon. In doing so, we provide insights into its evolution, global spread and potential role in diseases.

## 2 Methods

### 2.1 Transcriptome Mining

We employed the Serratus RdRp and palmID search tools to screen for ASCV‐related sequences within the NCBI SRA, using RdRp sequences of ASCV reference genomes available on NCBI/GenBank (YP_009026987 and AHX24377) (Babaian and Edgar 2022; Edgar et al. 2022). For the RdRp search, we used an alignment identity of 70% and a threshold score of 50. In addition, we screened the Transcriptome Shotgun Assembly (TSA) database using the ASCV reference genomes with BLASTN and TBLASTN (Zhang et al. 2000; Altschul et al. 1997). From these searches, sequencing projects that mapped to the ASCV reference sequences were selected for virome assembly.

### 2.2 Virome Assembly

We used an unbiased approach to allow for the characterisation of ASCV and other fish viruses that may be present. Raw sequence reads were quality trimmed using Trimmomatic (v.0.39) with the parameters SLIDINGWINDOW: 4:5, LEADING:5, TRAILING:5 and MINLEN:25 and then assembled into contigs using MEGAHIT (v.1.2.9) with default parameters (Bolger et al. 2014; Li and Dewey 2011). The resultant contigs were compared against the NCBI non‐redundant protein (nr) and nucleotide (nt) databases (August 2024) using DIAMOND (BLASTX) (v.2.0.9) and BLASTN (Buchfink et al. 2015; Zhang et al. 2000). Contigs that mapped to viral sequences were then predicted to open reading frames and used as queries for a second search against NCBI databases (nt/nr) using BLAST for validation in Geneious Prime (v.2024.0.5) (Kearse et al. 2012).

### 2.3 Genome Annotation

ASCV genomes were annotated using the NCBI conserved domain search tool and InterProScan with the TIGRFAMs (v.15.0), SFLD (v.4.0), PANTHER (v.15.0), SuperFamily (v.1.75), PROSITE (v.2022_01), CDD (v.3.18), Pfam (v.34.0), SMART (v.7.1), PRINTS (v.42.0) and CATH‐Gene3D databases (v.4.3.0) (Jones et al. 2014). Coverage was examined by mapping using Bowtie2 (v.2.3.3.1) (Langmead and Salzberg 2012). Coverage plots were constructed using the *ggplot2* package in R (v.4.4.0) (Ginestet 2011).

### 2.4 Phylogenetic Analysis

We constructed a family‐wide phylogeny of the *Caliciviridae* using amino acid sequences of the RdRp gene and a *Salovirus* phylogeny using nucleotide sequences of the virus polyprotein. Viral sequences were aligned with all related sequences on NCBI/GenBank using MAFFT (v.7.450) with the E‐INS‐i algorithm (Katoh and Standley 2013). Sequence alignments were trimmed using TrimAl (v.1.2) with a gap threshold of 0.9 and a variable conserve value (Capella‐Gutiérrez et al. 2009). To estimate the best‐fit model of amino acid/nucleotide substitution, we used the ModelFinder Plus (−m MFP) flag implemented in IQ‐TREE (v.1.6.12), and trees were estimated using a maximum likelihood approach with 1000 bootstrap replicates (Kalyaanamoorthy et al. 2017; Nguyen et al. 2015). Phylogenies were annotated using FigTree (v.1.4.4) (http://tree.bio.ed.ac.uk/software%20/figtree/).

### 2.5 Viral Abundance and Library Composition

Viral contig abundances were calculated using RNA‐Seq by Expectation Maximisation (RSEM) (v.1.3.0) (Li and Dewey 2011). We used the stably expressed fish gene—ribosomal protein S13 (RPS13)—as a reference marker (Geoghegan et al. 2021; Costa et al. 2023). We performed analysis of variance (ANOVA) to assess differences in viral abundance across ASCV genotypes, followed by a Tukey's post hoc test in R (v.4.4.0). Abundance plots were created using the *ggplot2* package (Ginestet 2011). To identify the presence of contamination, such as unexpected animal or human reads, raw sequence reads were mapped to a custom database containing all nucleotide sequences (excluding artificial or environmental sequences) on NCBI using KMA and CCMetagen (Clausen et al. 2018; Marcelino et al. 2020).

## 3 Results

### 3.1 Diversity and Abundance of ASCV in Chile, Scotland and Norway

**FIGURE 1:**
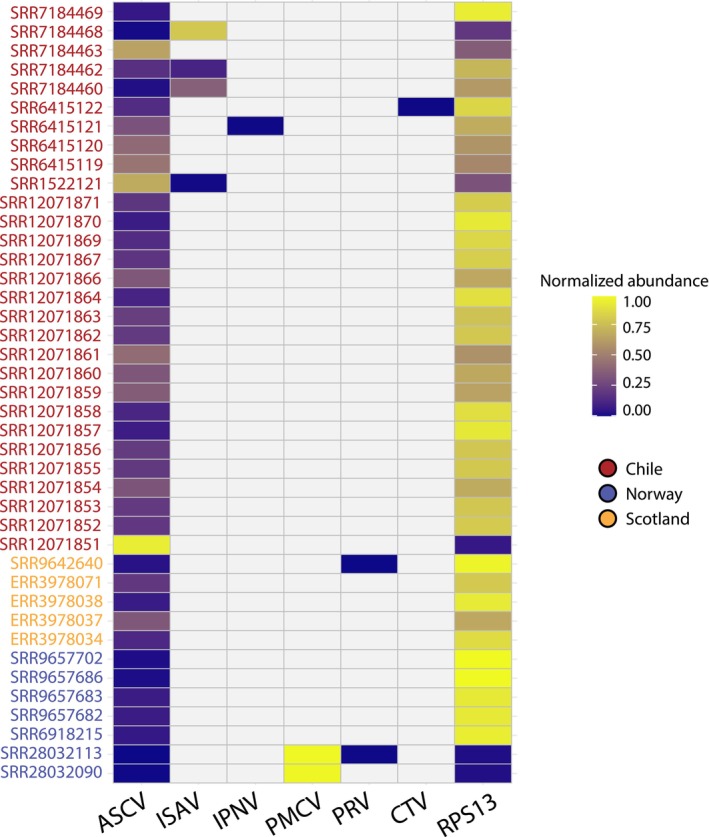
Abundance of ASCV and other viruses. Normalised viral and host (RPS13) abundance. Coloured IDs denote countries of origin.

We identified ASCV species in 41 RNA sequencing libraries on the SRA database. Of these, 29 were from experimental studies conducted in Chile, seven were from Norway (four experimental and three field samples) and five were from Scotland, including four field samples (Table 1). In addition to Atlantic salmon, we detected ASCV in wild common whitefish ( *Coregonus lavaretus* ) from Norway (Rougeux et al. 2019). ASCV RNA represented an average of 0.01% of the total reads in each library (range 0.000006%–0.05%) (Figure 1). Importantly, salmonid RNA accounted for an average of 99% of the total reads in each library, reflecting no contamination of unexpected animal taxa. We also detected reads from *Caligus rogercresseyi* and *Lepeophtheirus salmonis* —which are common parasites of salmon—comprising < 1% of the total reads.

**TABLE 1:** Host library information.

SRA ID	Country	Host	Tissue	NCBI bioproject	Source	Comments
SRR12071851	Chile	*S. salar*	Head kidney	PRJNA641181	Experimental	*P. salmonis* challenge
SRR12071852	Chile	*S. salar*	Head kidney	PRJNA641181	Experimental	*P. salmonis* challenge
SRR12071853	Chile	*S. salar*	Head kidney	PRJNA641181	Experimental	*P. salmonis* challenge
SRR12071854	Chile	*S. salar*	Head kidney	PRJNA641181	Experimental	*P. salmonis* challenge
SRR12071855	Chile	*S. salar*	Head kidney	PRJNA641181	Experimental	*P. salmonis* challenge
SRR12071856	Chile	*S. salar*	Head kidney	PRJNA641181	Experimental	*P. salmonis* challenge
SRR12071857	Chile	*S. salar*	Head kidney	PRJNA641181	Experimental	*P. salmonis* challenge
SRR12071858	Chile	*S. salar*	Head kidney	PRJNA641181	Experimental	*C. rogercresseyi* and *P. salmonis* challenge
SRR12071859	Chile	*S. salar*	Head kidney	PRJNA641181	Experimental	*P. salmonis* challenge
SRR12071860	Chile	*S. salar*	Head kidney	PRJNA641181	Experimental	*C. rogercresseyi* and *P. salmonis* challenge
SRR12071861	Chile	*S. salar*	Head kidney	PRJNA641181	Experimental	*C. rogercresseyi* and *P. salmonis* challenge
SRR12071862	Chile	*S. salar*	Head kidney	PRJNA641181	Experimental	*C. rogercresseyi* and *P. salmonis* challenge
SRR12071863	Chile	*S. salar*	Head kidney	PRJNA641181	Experimental	*C. rogercresseyi* and *P. salmonis* challenge
SRR12071864	Chile	*S. salar*	Head kidney	PRJNA641181	Experimental	*C. rogercresseyi* and *P. salmonis* challenge
SRR12071866	Chile	*S. salar*	Head kidney	PRJNA641181	Experimental	*C. rogercresseyi* and *P. salmonis* challenge
SRR12071867	Chile	*S. salar*	Head kidney	PRJNA641181	Experimental	*C. rogercresseyi* and *P. salmonis* challenge
SRR12071869	Chile	*S. salar*	Head kidney	PRJNA641181	Experimental	*P. salmonis* challenge
SRR12071870	Chile	*S. salar*	Head kidney	PRJNA641181	Experimental	*P. salmonis* challenge
SRR12071871	Chile	*S. salar*	Head kidney	PRJNA641181	Experimental	*P. salmonis* challenge
SRR1522121	Chile	*S. salar*	Pooled tissue	PRJNA255876	Experimental	ISAV challenge (Valenzuela‐Miranda, Boltaña, et al. 2015)
SRR6415119	Chile	*S. salar*	Head kidney	PRJNA422303	Experimental	*P. salmonis* challenge (Rozas‐Serri et al. 2018)
SRR6415120	Chile	*S. salar*	Head kidney	PRJNA422303	Experimental	*P. salmonis* challenge (Rozas‐Serri et al. 2018)
SRR6415121	Chile	*S. salar*	Head kidney	PRJNA422303	Experimental	Healthy (Rozas‐Serri et al. 2018)
SRR6415122	Chile	*S. salar*	Head kidney	PRJNA422303	Experimental	*P. salmonis* challenge (Rozas‐Serri et al. 2018)
SRR7184460	Chile	*S. salar*	Head kidney	PRJNA472087	Experimental	ISAV challenge (Valenzuela‐Miranda, Cabrejos, et al. 2015)
SRR7184462	Chile	*S. salar*	Head kidney	PRJNA472087	Experimental	ISAV challenge (Valenzuela‐Miranda, Cabrejos, et al. 2015)
SRR7184463	Chile	*S. salar*	Head kidney	PRJNA472087	Experimental	ISAV challenge (Valenzuela‐Miranda, Cabrejos, et al. 2015)
SRR7184468	Chile	*S. salar*	Gills	PRJNA472087	Experimental	ISAV challenge (Valenzuela‐Miranda, Cabrejos, et al. 2015)
SRR7184469	Chile	*S. salar*	Gills	PRJNA472087	Experimental	ISAV challenge (Valenzuela‐Miranda, Cabrejos, et al. 2015)
SRR9657682	Norway	*S. salar*	Head kidney	PRJNA527058	Experimental	Study evaluating immune response to feed (Andresen et al. 2019)
SRR9657683	Norway	*S. salar*	Head kidney	PRJNA527058	Experimental	Study evaluating immune response to feed (Andresen et al. 2019)
SRR9657686	Norway	*S. salar*	Head kidney	PRJNA527058	Experimental	Study evaluating immune response to feed (Andresen et al. 2019)
SRR9657702	Norway	*S. salar*	Head kidney	PRJNA527058	Experimental	Study evaluating immune response to feed (Andresen et al. 2019)
ERR3978034	Scotland	*S. salar*	Gills	PRJEB37113	Field	Transcriptome study on proliferative gill disease (Krol et al. 2020)
ERR3978037	Scotland	*S. salar*	Gills	PRJEB37113	Field	Transcriptome study on proliferative gill disease (Krol et al. 2020)
ERR3978038	Scotland	*S. salar*	Gills	PRJEB37113	Field	Transcriptome study on proliferative gill disease (Krol et al. 2020)
ERR3978071	Scotland	*S. salar*	Gills	PRJEB37113	Field	Transcriptome study on proliferative gill disease (Krol et al. 2020)
SRR9642640	Scotland	*S. salar*	Head kidney	PRJNA552604	Experimental	Transcriptome study on amoebic gill disease (Robledo et al. 2020)
SRR6918215	Norway	*C. lavaretus*	Liver	PRJNA448004	Field	Study evaluating the *Coregonus* species complex (Rougeux et al. 2019)
SRR28032113	Norway	*S. salar*	Heart	PRJNA1064402	Field	PMCV genomic sequencing from CMS field cases (Amono et al. 2024)
SRR28032090	Norway	*S. salar*	Heart	PRJNA1064402	Field	PMCV genomic sequencing from CMS field cases (Amono et al. 2024)

Among ASCV‐positive libraries, 76% were detected in the head kidney, followed by gills (15%), heart (5%), liver and pooled tissues (both 2%) (Table 1). Alongside ASCV, our metatranscriptomic analysis identified the presence of other viruses such as infectious salmon anaemia virus (ISAV), infectious pancreatic necrosis virus (IPNV), piscine myocarditis virus (PMCV), piscine orthoreovirus (PRV) and cutthroat trout virus (CTV) (Figure 1; Table S1). The vast majority of these were already described in the parent study (Table 1).

### 3.2 Phylogenetic Relationships of Novel ASCV Sequences

**FIGURE 2:**
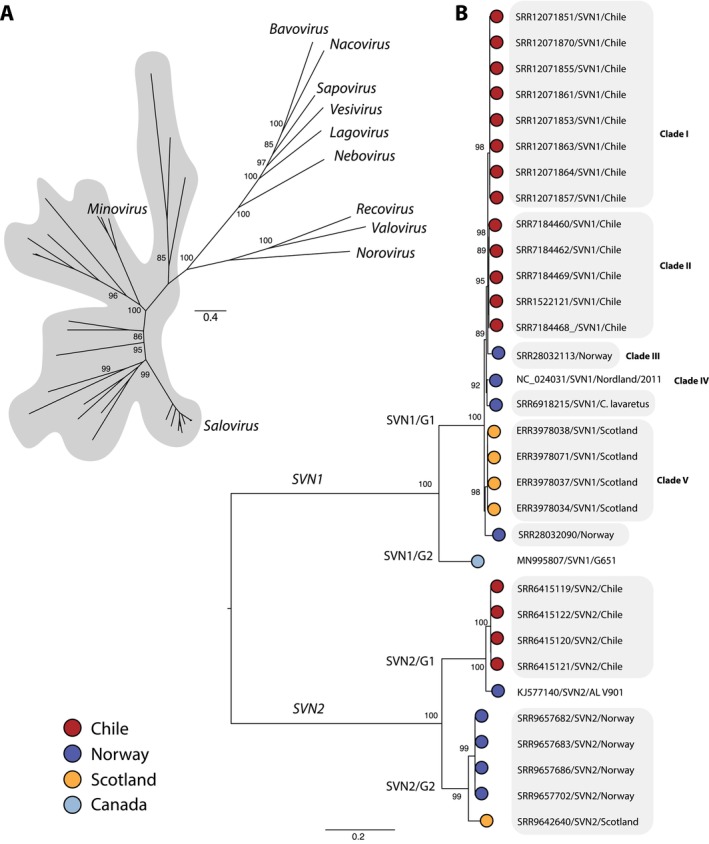
Phylogenetic relationships among caliciviruses. (A) Unrooted phylogeny of the *Caliciviridae* using amino acid sequences of the RdRp gene. Scale bar represents amino acid substitutions per site. Shaded taxa denote fish caliciviruses. Unlabelled tips represent unclassified fish caliciviruses. Scale bar represents amino acid substitutions per site. (B) Phylogeny of the genus *Salovirus*, estimated using a nucleotide alignment of the viral polyprotein. In cases where identical sequences were identified (i.e., from the same NCBI BioProject), the largest viral contig was selected for phylogenetic analysis. Shaded taxa represent viruses discovered in this study. The tree was midpoint rooted for clarity only. Scale bar represents nucleotide substitutions per site.

**FIGURE 3:**
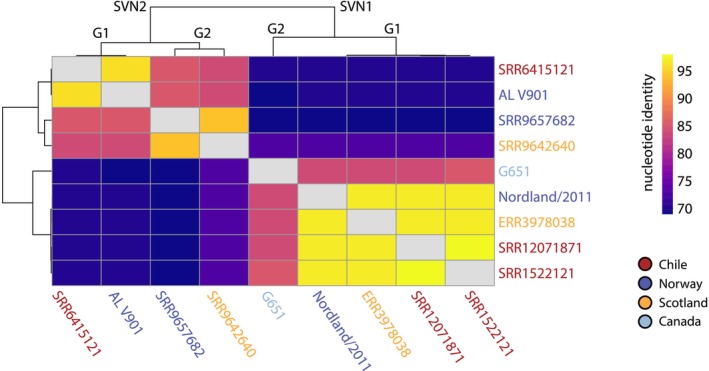
Percentage identity matrix of salovirus species. Matrix constructed using complete genomes of SVN1/G1 and G2, and SVN2/G1 and near‐complete genomes of SVN2/G2.

We constructed a family‐wide phylogeny of the *Caliciviridae*, which separated saloviruses from other fish‐infecting caliciviruses (Figure 2A). A notable observation from these data was that ASCV constitutes two divergent viral species, which we provisionally name *Salovirus nordlandense 1* and *2* (SVN1 and 2), exhibiting ~70% nucleotide similarity (Figure 2B; Figure 3).

We identified SVN1 in 32 sequencing libraries that can be classified into two genotypes. Genotype 1 incorporates five clades. Clades I and II comprise viruses from *S. salar* in Chile, illustrating a geographic signal. Similarly, viruses infecting *S. salar* and *C. lavaretus* in Norway fell within Clades III and IV, while viruses in *S. salar* from both Scotland and Norway clustered in Clade V. All members of genotype 1 had 96.5% nucleotide identity across the entire genome (Figures 2B and 3). Genotype 2 contained one virus found only in Canada, exhibiting ~85% nucleotide similarity with genotype 1.

In a similar manner, we identified two genotypes of SVN2 that were spread across Chile, Norway and Scotland. It was notable that we detected close relatives (~97% nucleotide identity) of the cell culture isolate of ASCV (AL V901; Mikalsen et al. 2014) in Chile and not Norway (SVN2/G1). SVN2 viruses from Norway fell within genotype 2, which also included a related virus found in Scotland.

We performed ANOVA to test for any differences in viral abundance between SVN1 and SVN2 genotypes. This revealed a significantly higher abundance of SVN2/G1 compared to SVN1/G1 (Tukey: *z* = 2.343, *p =* 0.048) and SVN2/G2 (Tukey: *z* = 2.75, *p* = 0.015) (Figure S1).

### 3.3 Genome Organisation and Coverage

**FIGURE 4:**
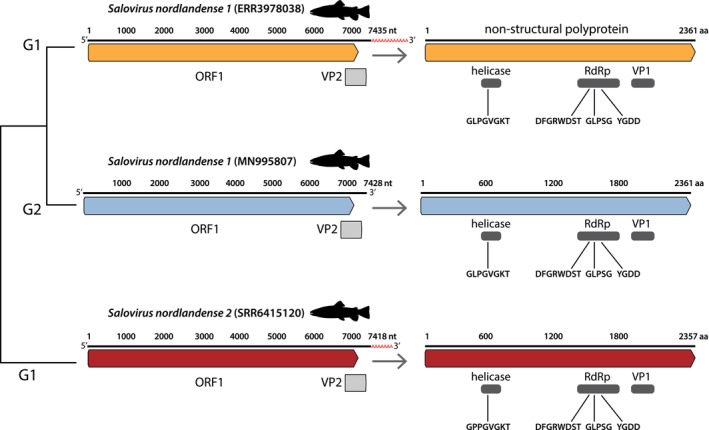
Organisation of SVN1 and SVN2 genomes. Genomes are displayed in the 5′‐3′ orientation. Arrows indicate translation of the ORF1 polyprotein, illustrating conserved domains and *Caliciviridae* motifs. Genomes for each viral species and genotype were chosen based on contig size (i.e., complete genomes). Partial genomes of SVN2 genotype 2 viruses are not displayed. Tree labels denote genotypes: G1 = genotype 1 and G2 = genotype 2.

**FIGURE 5:**
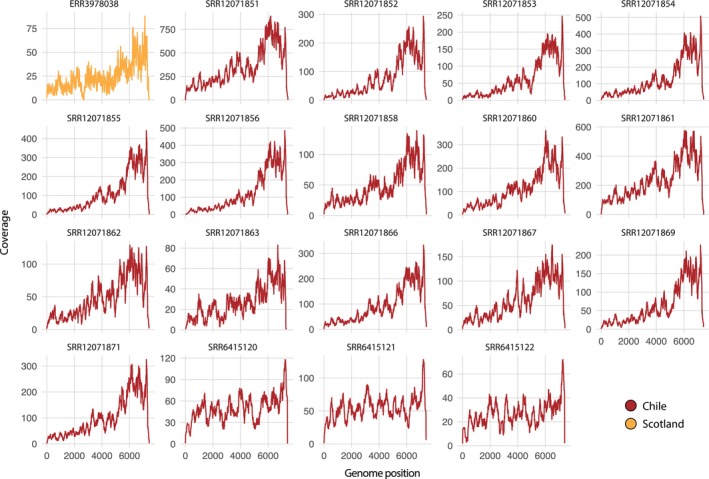
Genome coverage. The *x* axis denotes genome position (see Figure 4) and the *y* axis represents coverage. Genomes are displayed in the 5′‐3′ orientation.

We identified complete genomes in 19 sequencing libraries. Both SVN1 and SVN2 exhibited typical genome structure as described in Mikalsen et al. (2014) (Figure 4). Both SVN1 genotypes contained conserved *Caliciviridae* motifs, comprising a helicase motif (GLPGVGKT) and RdRp motifs (DFGRWDST, GLPSG and YGDD). The motif sequences were highly similar in SVN2; however, the size of ORF1 was four amino acids shorter, and ORF2 was one amino acid shorter (Figure 4). All viral genomes exhibited an average of 94× coverage in each transcriptome. It was notable that we identified higher genome coverage in the VP1–VP2 region, which was consistent across all viral genomes (Figure 5).

## 4 Discussion

Screening of publicly available transcriptomes led to the identification of ASCV in Chile and Scotland as well as the presence of novel genotypes in Norway. Our phylogenetic analysis confirmed that ASCV comprises two viral species, which we provisionally name SVN1 and SVN2. It was notable that we identified a broad distribution of all viral genotypes, with no clear geographic pattern: both viral species were found in Chile, Scotland and Norway. The evolutionary divergence of the *Salovirus* genus from other fish caliciviruses, along with its strong association with salmonids, indicates that this viral group commonly infects salmonid fish. For instance, while we screened NCBI databases, we only identified SVN1 and SVN2 in salmonids, such as Atlantic salmon and common whitefish, and SVN1 has also been detected in Pacific salmon in Canada (Mordecai et al. 2021).

It was notable that Atlantic salmon in Chile served as a rich source of SVN1 and SVN2 diversity, clustering with viruses from Norway in the viral phylogeny. Atlantic salmon was introduced to Chile primarily through the importation of eyed eggs from Norway, Scotland and Canada, as well as the transportation of live fish (Bjørndal and Aarland 1999). In 2007, following the emergence of ISAV in Chile, which had large socioeconomic and environmental impacts, the government implemented stringent biosecurity measures to mitigate the risk of emerging infectious diseases. Currently, Chilean farms can only import eyed eggs from Iceland (Moncayo et al. 2024). Similarly, there are strict regulations governing the importation of live individuals (SERNAPESCA 2020).

This regulatory shift in Atlantic salmon importation suggests that SVN1 and SVN2 were likely introduced to Chile during the early stages of Atlantic salmon farming through a combination of both vertical transmission (eggs) and horizontal transmission (live fish). The possibility of vertical transmission is supported by our identification of both viral species in experimental fish that were likely not exposed to field conditions or pathogens (Table 1). Furthermore, the detection of SVN1 in wild common whitefish in Norway and wild chinook salmon in Canada provides additional evidence for the potential role of horizontal transmission (Mordecai et al. 2021).

Given the presence of SNV1 and SNV2 in both healthy and diseased fish, their role in disease remains unclear, despite evidence that SNV2 can cause systemic infection in Atlantic salmon in vivo (Mikalsen et al. 2014). For example, we identified SVN1 in apparently healthy experimental Atlantic salmon (Andresen et al. 2019). It was notable that we identified close relatives of the SVN2 cell culture isolate—AL V901—in Chile, which we provisionally classified as SVN2/G1 (Mikalsen et al. 2014). This genotype exhibited significantly higher viral abundance than other genotypes, along with a smaller genome size. The novel SVN2/G1 viruses identified from Chile were part of a study that investigated transcriptomic profiles in response to *P. salmonis* (Rozas‐Serri et al. 2018). Therefore, it is unclear whether and how SVN2/G1 played a role in disease.

Of note was the discovery of SVN1/G1 in *S. salar* suffering from proliferative gill disease (PGD) in Scotland: a syndrome characterised by histological proliferation and thought to be caused by interacting pathogens and the environment (Herrero et al. 2018; Krol et al. 2020). However, the exact role of these pathogens in PGD remains unclear, and it is possible that SVN1/G1 might represent a novel component cause. Indeed, the parent study (Krol et al. 2020), which analysed differentially expressed genes between salmon with low gill pathology and those with moderate gill pathology (SVN1‐positive libraries), identified interferon gamma—an antiviral protein of the adaptive immune system—as one of the top upstream regulators in salmon with higher gill pathology. This suggests that SVN1/G1 might play a role in PGD (Krol et al. 2020). Indeed, Mikalsen et al. (2014) identified the presence of ASCV in gill tissue in 100% of samples after 8 weeks post challenge and in 50% of samples after 16 weeks.

As such, we hypothesise that SVN1 and SVN2 might be frequently associated with co‐infections. This is supported by the fact that 88% of the libraries screened in this study were initially examined for infectious diseases or multifactorial diseases in addition to the viruses detected through our metatranscriptomic analysis (Table 1). Moreover, it was notable that some libraries exhibited a higher abundance of ASCV than the pathogen of original interest (Figure 1). For instance, the SVN1 library from Chile (SRA: SRR1522121) showed a higher abundance of ASCV than ISAV, despite the fish being challenged with ISAV to assess transcriptional responses. Indeed, the first discovery of ASCV (SVN1/G1) was identified alongside PRV in *S. salar* suffering from heart and skeletal muscle inflammation (Mikalsen et al. 2014). While the precise mechanisms of SNV1/SVN2 infection are unclear, it is notable that other caliciviruses have been associated with co‐infections, including fathead minnow calicivirus (Mor et al. 2017). In addition, feline calicivirus often acts as a secondary agent to Felid herpesvirus‐1 in kittens with pneumonia (Rodriguez et al. 2018).

It is important to note that this study screened publicly available transcriptome data, which may introduce biases due to the quality of the data sets, as well as the lack of control over experimental conditions and contamination. As such, our results reflect taxonomic associations of ASCV among salmonid populations rather than definitive evidence of infection and transmission. However, our ability to retrieve complete genomes, along with continuous genome coverage and a high abundance of expected salmonid host reads, strongly suggests that the viruses in question were associated with active infection. For instance, higher levels of coverage were consistently observed in the VP1–VP2 region. Given that the feline calicivirus VP2 protein has been shown to be crucial for the synthesis and maturation of infectious virions for other caliciviruses (Sosnovtsev et al. 2005), these data support the idea that the viruses were likely involved in infection at the time of sampling. The VP2 protein has also been proposed to function as a channel, enabling the delivery of the feline calicivirus genome into the host cell's cytoplasm, thus contributing to the initial stages of infection (Conley et al. 2019).

Taken together, our findings illustrate the widespread distribution and extensive genetic diversity of SVN1 and SVN2 in both experimental and field (aquaculture) populations of Atlantic salmon in Chile, Scotland and Norway, suggesting that aquaculture has played a role in shaping the evolution and ecology of these viruses. As such, future work should examine whether ASCV can be vertically transmitted and persist in eyed eggs, and evaluate the efficacy of current sterilisation methods in preventing viral transmission. This is of particular importance for assessing the health status of experimental animals, especially those used for vaccine development. While SVN2 has been shown to cause systemic infection in vivo, further studies are required to understand the mechanisms of infection, how it differs between SVN1 and SVN2 and whether these viruses contribute to co‐infections and multifactorial diseases. Additionally, further sequencing projects in other major salmon farming regions are needed to fully understand their global spread.

## Author Contributions

**Vincenzo A. Costa:** conceptualization, investigation, writing – original draft, methodology, writing – review and editing, visualization, formal analysis. **Aase B. Mikalsen:** conceptualization, writing – review and editing, methodology. **Francisca Samsing:** conceptualization, funding acquisition, writing – review and editing.

## Conflicts of Interest

The authors declare no conflicts of interest.

## Supporting information


**Figure S1.** Comparison of viral abundance between ASCV genotypes. Asterisk denotes significant differences between groups (*p = < 0.05).

**Table S1.** Description of the viruses identified in this study.

## Data Availability

The newly described viral genomes have been deposited in NCBI/GenBank under the accessions BK069822–BK069827.
