# Supplementary figures and images for: Phylogenetic Diversity and Geographic Distribution of Atlantic Salmon Calicivirus in Major Salmon Farming Regions
Source: J Fish Dis. 2025 Feb 19;48(6):e14107. doi: 10.1111/jfd.14107 (PMC12068843; doi:10.1111/jfd.14107)

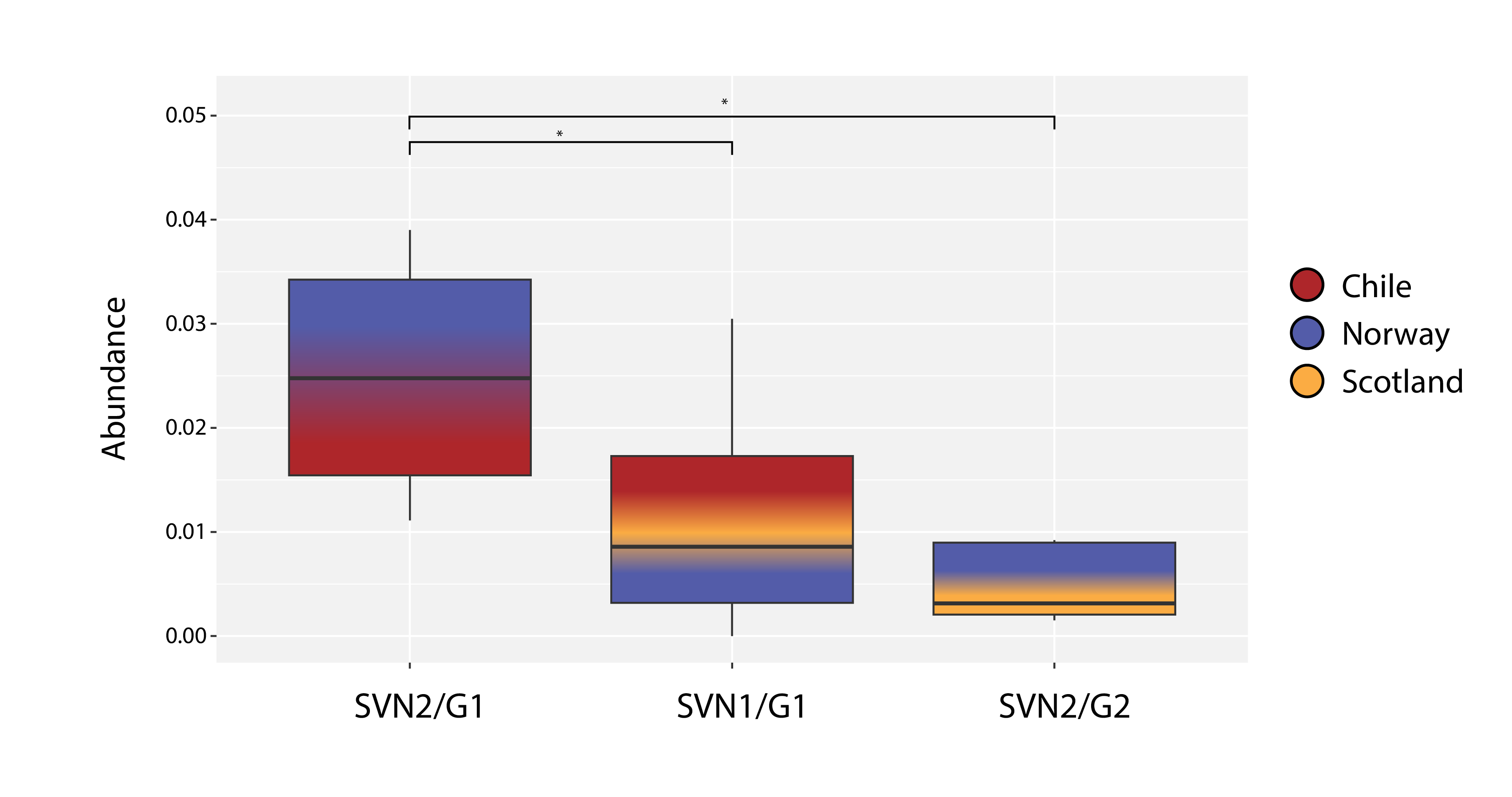

Supplement: Supplementary file 1 — Figure S1. Comparison of viral abundance between ASCV genotypes. Asterisk denotes significant differences between groups (*p = < 0.05). [file JFD-48-e14107-s001.tif]
